# Supplementary material for: The Biological Significance of Pyruvate Sensing and Uptake in Salmonella enterica Serovar Typhimurium
Source: Microorganisms. 2022 Aug 30;10(9):1751. doi: 10.3390/microorganisms10091751 (PMC9504724; doi:10.3390/microorganisms10091751)
Supplement: Supplementary file 1 [file microorganisms-10-01751-s001.zip › microorganisms-1839009-supplementary.pdf]

Supplementary material

The biological significance of pyruvate sensing and uptake in *Salmonella enterica* serovar Typhimurium

Stephanie Paulini [1], Florian D. Fabiani [1,†], Anna S. Weiss [2], Ana Laura Moldoveanu [3], Sophie Helaine [3,‡], Bärbel Stecher [2,4] and Kirsten Jung [1,*]

[1] Department of Microbiology, Ludwig-Maximilians-University Munich, 82152 Planegg-Martinsried, Germany
[2] Max von Pettenkofer Institute of Hygiene and Medical Microbiology, Faculty of Medicine, Ludwig-Maximilians-University Munich, 80336 Munich, Germany
[3] MRC Centre for Molecular Bacteriology and Infection, Imperial College London, London SW7 2DD, UK
[4] German Center for Infection Research (DZIF), Partner Site LMU Munich, 80337 Munich, Germany
* Correspondence: jung@lmu.de; Tel.: +49-(0)89/2180-74500
† Present affiliation: Bayer AG, 13353 Berlin, Germany.
‡ Present affiliation: Department of Microbiology, Harvard Medical School, Boston, MA 02115, USA.

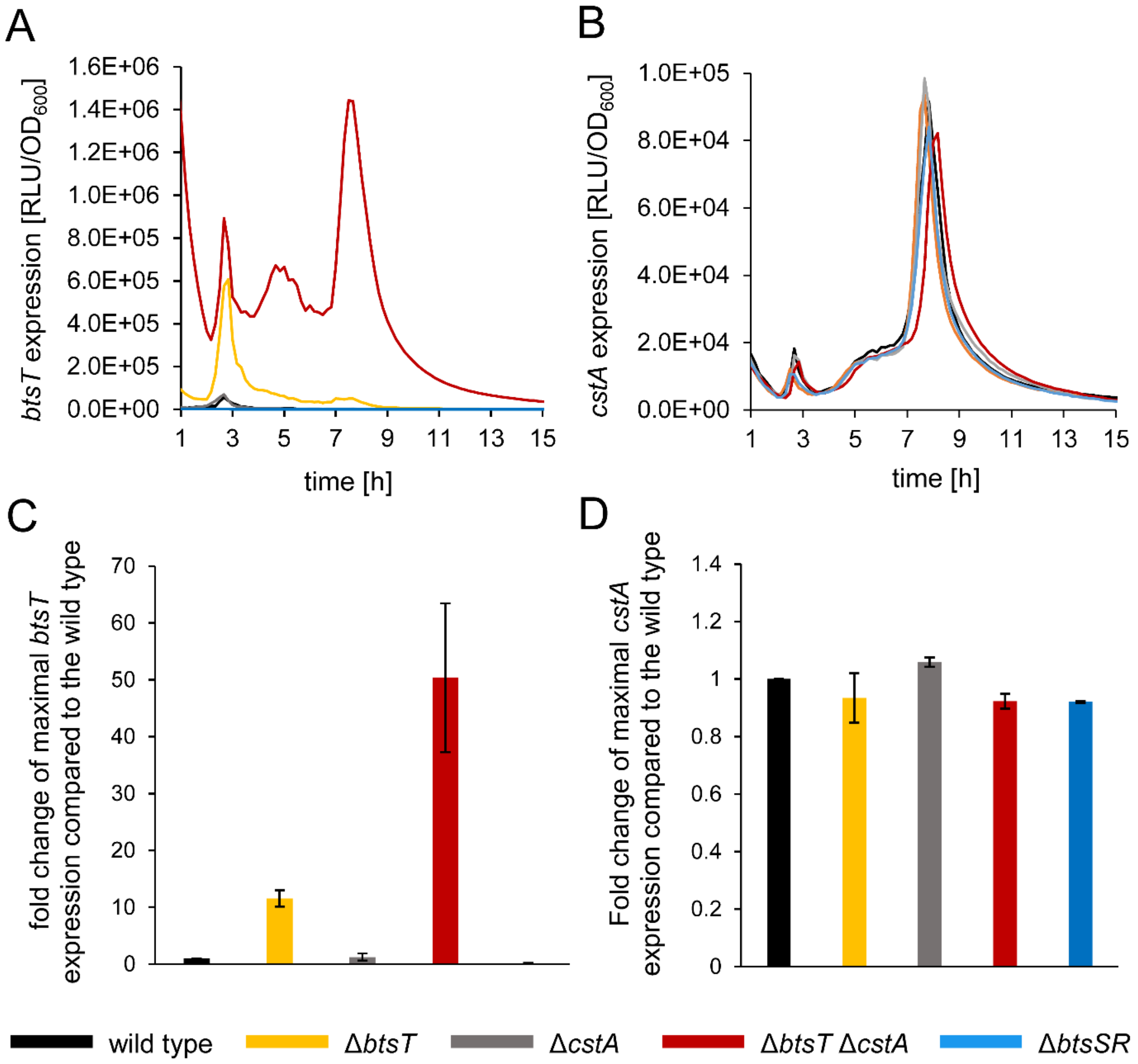

Figure S1. Expression of *btsT* and *cstA* in *S*. Typhimurium mutants. SL1344 wild-type (black), Δ*btsT* (yellow), Δ*cstA* (grey), Δ*btsT*Δ*cstA* (red) and Δ*btsSR* (blue) cells harboring the reporter plasmid for *btsT* (pBBR1-MCS5-*PbtsT-lux*) or for *cstA* (pBBR1-MCS5-*PcstA-lux*) were grown in LB medium in a plate reader at 37 °C. Luminescence values were measured over time and gene expression was determined as RLU per 1 OD_{600}. **A)** Expression of *btsT* during growth. **B)** Expression of *cstA* during growth. **C)** Maximal *btsT* expression depicted as fold change from wild-type value. **D)** Maximal *cstA* expression depicted as fold change from the wild-type value. A, B: graphs represent the mean of three independent replicates. the standard deviations were below 10%. C, D: Error bars represent the standard deviations of the mean of three independent replicates.

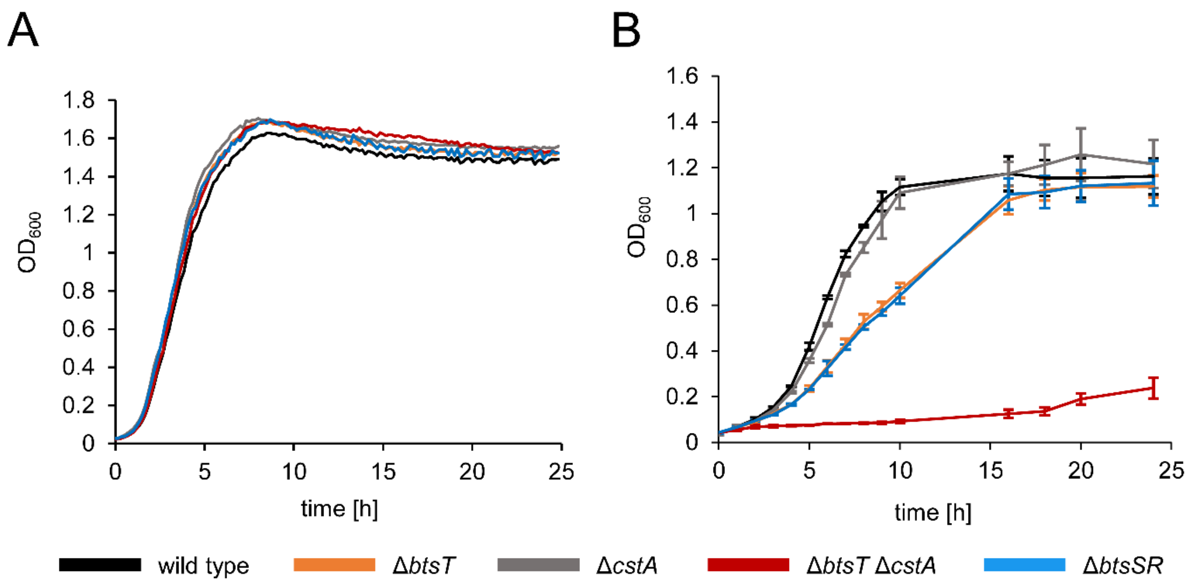

Figure S2. Growth of *S.* Typhimurium mutants. SL1344 wild-type (black), Δ*btsT* (yellow), Δ*cstA* (grey), Δ*btsT*Δ*cstA* (red) and Δ*btsSR* (blue) cells were grown for 24 hours at 37°C in **A)** LB medium or **B)** M9 minimal medium with 60 mM pyruvate.

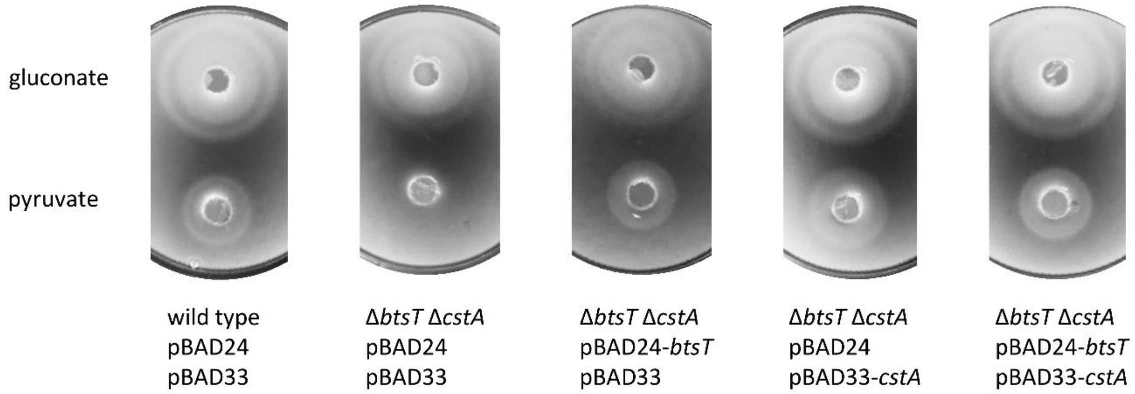

Figure S3. *S*. Typhimurium Δ*btsT*Δ*cstA* mutant lost chemotactic response to pyruvate. Chemotaxis was tested by mixing SL1344 wild-type or Δ*btsT*Δ*cstA* cells harboring the indicated expression plasmids for *btsT* (pBAD24-*btsT*) and/or *cstA* (pBAD33-*cstA*) or the empty vectors with 0.3% (wt/vol) M9 soft agar and pouring them over 1.5% (wt/vol) M9 agar plugs containing either 60 mM gluconate (above) or 60 mM pyruvate (below). Plates were incubated at 37 °C for 4 hours, and the pictures are representative of three independent experiments.

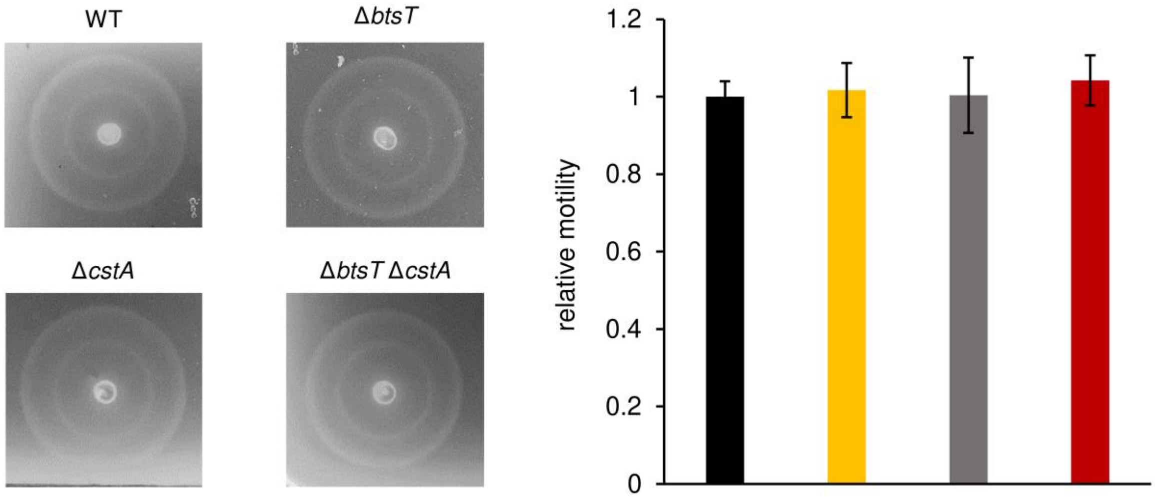

Figure S4. Motility of *S*. Typhimurium is not affected by deletions of *btsT* or *cstA*. Motility of SL1344 wild type (upper left, black), Δ*btsT* (upper right, yellow), Δ*cstA* (lower left, grey) and Δ*btsT*Δ*cstA* (lower right, red) cells was tested by spotting equal numbers of cells on 0.3% LB soft agar, incubating the plates at 37°C for 3 hours and measuring the diameter of the ring with the software ImageJ [54]. Images of rings are representative of four independent experiments and relative motility was determined in relation to the mean diameter of the wild type ring.

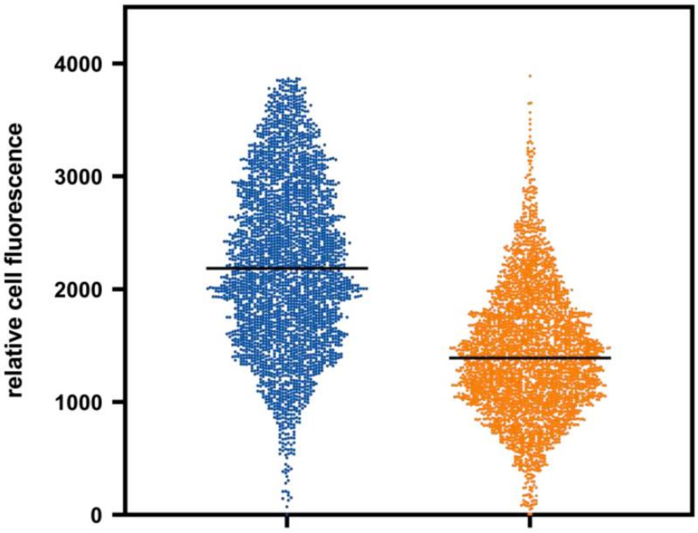

Figure S5. Expression of *btsT* in *S*. Typhimurium under SPI2 inducing conditions. Strain SL1344 *btsT*::*mNeonGreen,* which chromosomally encodes a fusion between *btsT* and *mNeonGreen,* was used to measure expression of *btsT* in single cells. Cells were grown in NonSPI2 (blue) or InSPI2 (orange) medium with 4 µg/ml histidine and 60 mM pyruvate as C-source until mid-exponential phase. Samples were taken for fluorescence microscopy. To quantify relative fluorescence intensities of single cells, phase contrast and fluorescent images were analyzed using the ImageJ [54] plugin MicrobeJ [77]. In total >1000 cells were quantified per condition.

Table S1. Oligonucleotides used in this study.

	DNA sequence	Description
#1	CGCTGTGCGCTACCCGGCATCAGTTTTAGTGGTGTGAAGATTAAGACCCACTTTCACATT	Primer 1 for tetRA-insertion by λ-Red recombination in *btsT*
#2	ATTAAACTTACAACCAGGTTTTACTATGGATACGAAAAAGCTAAGCACTTGTCTCCTG	Primer 2 for tetRA-insertion by λ-Red recombination in *btsT*
#3	ATTAAACTTACAACCAGGTTTTACTATGGATACGAAAAAGTCTTCACACCACTAAAACTG	Primer 1 for clean deletion of btsT
#4	AGTCCGGAATACCAATCAACA	Primer 2 for clean deletion of btsT
#5	ACCGCTTAAACCGCCATACA	Primer 1 for sequencing of Δ*btsT*
#6	ACGTTCGCGGAAGAACTCTT	Primer 2 for sequencing of Δ*btsT*
#7	GGCAAAACGATATTCTAACAGTCTTTTACAGGCCAATCGCTTAAGACCCACTTTCACATT	Primer 1 for tetRA-insertion by λ-Red recombination in *btsSR*
#8	TTTAATTGAAGTGTGGTTTGCGGGTATGTACGAGTTTAATCTAAGCACTTGTCTCCTG	Primer 2 for tetRA-insertion by λ-Red recombination in *btsSR*
#9	TTGTTGATACGACGTTCCGC	Primer 1 for clean deletion of btsSR
#10	TTTAATTGAAGTGTGGTTTGCGGGTATGTACGAGTTTAATGCGATTGGCCTGTAAAAGAC	Primer 2 for clean deletion of btsSR
#11	TGGAACACCCAAACGGACAACAACTATGAATAAATCAGGGTAGGCTGGAGCTGCTTCGAA	Primer 1 to amplify the FRT-kanamycin-FRT cassette from pKD46 for replacement of *cstA*
#12	GGAGAGGGCTATTGATGTAAAAAGATTAGTGCGCGCCTTTTCCTCCTTAGTTCCTATTCC	Primer 2 to amplify the FRT-kanamycin-FRT cassette from pKD46 for replacement of *cstA*
#13	CTCTTTGACGAGCAGGGGAG	Primer 1 for sequencing of Δ*cstA*
#14	CGTCTGATCCGGATGCGTTA	Primer 2 for sequencing of Δ*cstA*
#15	AAAAAATCTAGAGCGATGACGTGCTGGAGGCG	Primer 1 to amplify the promoter of *cstA* to create pBBR1-MCS5-*PcstA-lux*, XbaI site
#16	AAAAAACTCGAGAGTTGTTGTCCGTTTGGGTG	Primer 2 to amplify the promoter of *cstA* to create pBBR1-MCS5-*PcstA-lux*, XhoI site
#17	AAAAAATCTAGAAGTTTGCAATACGGTGAAGT	Primer 1 to amplify the promoter of *btsT* to create pBBR1-MCS5-*PbtsT-lux*, XbaI site
#18	AAAAAACTCGAGAGTAAAACCTGGTTGTAAGT	Primer 2 to amplify the promoter of *btsT* to create pBBR1-MCS5-*PbtsT-lux*, XhoI site
#19	GCGCGCGAATTCACTATGGATACGAAAAAGATATT	Primer 1 to amplify *btsT* to create pBAD24-*btsT*, EcoRI site

#20	GCTAGCAAGCTTTTAGTGATGGTGATGG TGATGGTGGTGTGAAGAGATCTTCA	Primer 2 to amplify *btsT* to create pBAD24-*btsT*, HindIII site
#21	GCGCGCGAATTCACTATGAATAAATCAG GGAAATA	Primer 1 to amplify *cstA* to create pBAD3-*cstA*, EcoRI site
#22	GCTAGCAAGCTTTTAGTGATGGTGATGG TGATGGTGCGCGCCTTTCGCCTGCG	Primer 2 to amplify *cstA* to create pBAD3-*cstA*, HindIII site

Supplementary methods

Strain construction of SL1344 *btsT*::*mNeonGreen*. Chromosomal fusions were created by double homologous recombination using the pNPTS138-R6KT suicide plasmid [47]. SL1344 *btsT* was amplified by PCR from SL1344 genomic DNA with oligonucleotides #38 and #39, without keeping the stop codon. The *mNeonGreen* gene was amplified by PCR from a plasmid (Peter Graumann, Marburg) with oligonucleotides #40 and #41. An 800 bp region directly downstream of *btsT* was amplified by PCR from SL1344 genomic DNA with oligonucleotides #42 and #43. The pNPTS138-R6KT backbone was linearized with oligonucleotides #37 and #44. All fragments were created with overlaps of 20 bp for assembly using the NEBuilder kit (New England Biolabs, Ipswich, MA, USA). The final pNPTS138-R6KT-*btsT*::*mNeonGreen* plasmid was first transformed into *E. coli* DH5α and confirmed by sequencing. Then it was transferred into *E. coli* WM3064 for conjugation with SL1344. Double homologous recombination was induced as described in the main manuscript. Oligonucleotide sequences are listed in table S1.

***In vivo* single cell fluorescence measurements.** SL1344 cells with the chromosomal fusion *btsT*::*mNeonGreen* were grown in InSPI2 and NonSPI2 medium [69] with 4 µg/ml histidine and 60 mM pyruvate as carbon source, inoculated from overnight culture to an initial OD_{600} of 0.05. In exponential growth phase, samples were taken and 2 µl of the culture were spotted on 1% (wt/vol) agarose prepared with PBS on a microscope slide and sealed with a cover slide. Microscopy was performed using a DMI6000 B fluorescence microscope (Leica Microsystems, Wetzlar, Germany), with an excitation wavelength of 485 nm and a 510-nm emission filter. To quantify single cell fluorescence, 1000 cells per condition (InSPI2 or NonSPI2) were analyzed using the plug-in MicrobeJ [77] of the software ImageJ [54], as described before [46]. The background was subtracted for each cell.
